# The impact of *Bacillus subtilis* DSM32315 and L-Threonine supplementation on the amino acid composition of eggs and early post-hatch performance of ducklings

**DOI:** 10.3389/fvets.2023.1238070

**Published:** 2023-08-23

**Authors:** Mahmoud Mostafa Azzam, Wei Chen, Weiguang Xia, Shuang Wang, Yanan Zhang, HebatAllah Kasem El-Senousey, Chuntian Zheng

**Affiliations:** ^1^Animal Production Department, College of Food and Agriculture Sciences, King Saud University, Riyadh, Saudi Arabia; ^2^Guangdong Academy of Agricultural Sciences, Key Laboratory of Animal Nutrition and Feed Science (South China) of Ministry of Agriculture, State Key Laboratory of Swine and Poultry Breeding Industry, Guangdong Public Laboratory of Animal Breeding and Nutrition, Guangdong Key Laboratory of Animal Breeding and Nutrition, Institute of Animal Science, Guangzhou, China; ^3^Department of Animal Production, Faculty of Agriculture, Cairo University, Giza, Egypt

**Keywords:** ducks, Threonine, *Bacillus subtilis*, amino acid composition of eggs, hatch weight of ducklings

## Abstract

Poultry requires Threonine, an essential amino acid, and its metabolites for proper metabolic function. Threonine is crucial in the biosynthesis of mucin, which is essential for intestinal health and nutrient absorption. *Bacillus subtilis* (*B. subtilis*) is a potential substitute for antibiotic growth promoters in the poultry industry. The current study was designed to evaluate the simultaneous effect of L-Threonine (Thr) and *B. subtilis* DSM32315 supplementation on laying duck breeders in order to maximize performance. A total number of 648 female 23-week-old Longyan duck breeders were assigned to a 3 × 2 factorial design with six replicates of 18 birds per replicate. L-Thr was added to the control diet at concentrations of 0, 0.7, and 1.4 g/kg, equating to 3.9, 4.6, and 5.3 g Thr/kg, with or without *B. subtilis* strain DSM 32315 (0.0 and 0.5 g/kg). Increasing Thr concentrations improved egg production and ducklings’ hatchling weight (*p* < 0.05). In addition, L-Thr supplementation resulted in a tendency for decreased feed conversion ratio without affecting egg quality. There was no significant effect (*p* > 0.05) of the dietary Thr levels on egg yolk and albumen amino acid concentrations. In contrast, the addition of *B. subtilis* decreased the concentrations of amino acids, excluding proline, in the egg white (albumen) and the egg yolk (*p* < 0.05). Furthermore, the supplementation of *B. subtilis* decreased (*p* < 0 0.001) the hatching weight of ducklings. The addition of *B. subtilis* without L-Thr decreased (*p* < 0.05) the hatchability of fertile eggs and the hatching weight of ducklings compared to those of ducks fed dietary L-Thr along with *B. subtilis* (*p* < 0.001). The combining L-Thr at 0.7 g/kg with *B. subtilis* DSM 32315 at 0.5 g/kg could increase eggshell quality, hatchability, and hatching weight. The current study revealed that the combination supplemented of L-Thr and *B. subtilis* DSM 32315 is recommended due to its positive effects on the eggshell percentage, hatchability and the body weights of newly hatched ducklings when dietary Thr was added at a rate of 0.7 g/kg and *B. subtilis* DSM 32315 at 0.5 g/kg. In addition, adding L-Thr separately at 0.7 g/kg could improve the egg production of duck breeders. Further studies are required to find the proper dosages of *B. subtilis* DSM 32315 with co-dietary inclusion of limiting amino acids in the diets of duck breeders. The findings of these trials will support feed additive interventions to transition into antibiotic-free diets.

## 1. Introduction

The use of antibiotic growth promoters (AGP) has been banned in poultry farms worldwide. Therefore, global efforts in the poultry industry are focused on the development of effective feeding strategies that provide comparable benefits. *Bacillus subtilis* DSM 32315 has been effectively utilized as a substitute for AGP in broiler feeding (1–3). *Bacillus subtilis* DSM 32315 has unique beneficial effects on broilers; dietary supplementation of *B. subtilis* DSM 32315 improved the growth performance of birds and the intestinal microflora balance (4). The addition of *B. subtilis* to laying hens’ diets increased egg production, health, and egg quality’ (5, 6). Furthermore, the application of dietary *B. subtilis* (C-3102) improved the egg weight, fertility and hatchability, and eggshell thickness in laying hen breeders (7). *Bacillus subtilis* supplementation has a significant impact on the levels of a wide range of chemical metabolites in the chicken intestine, particularly those related to amino acids (8). Moreover, adding a probiotic to an amino acid supplement has been demonstrated to have a synergistic or additive effect on productivity, egg quality, and immunity (9). Therefore, the combination of Thr (an essential amino acid) and probiotic *Bacillus* may increase the absorptive area and functional ability of the intestine, thereby increasing production and egg quality.

Threonine is an essential amino acid for poultry because it is the third most limiting amino acid, and its metabolites are necessary for the metabolic process. Thr is essential for mucin biosynthesis, which is crucial for intestinal health and the absorption of nutrients. Previous research has demonstrated that Thr supplementation significantly increases egg production in laying ducks and breeder hens (10–12). However, previous studies mainly focused on the effects of supplementing *B. subtilis* DSM 32315 as a potential probiotic in diets as individual supplements. To our knowledge, no prior study has evaluated the combined effects of dietary Thr and *B. subtilis* on laying duck breeders, as far as we are aware. Consequently, this study aimed to investigate the interactive effects of dietary Thr and *B. subtilis* on laying duck breeders’ reproductive performance, the eggs’ amino acid composition, and early post-hatch duckling performance’.

## 2. Materials and methods

### 2.1. Experimental design and dietary treatments

In a randomized block design, six hundred and forty-eight of female Longyan duck breeders, with comparable body weight (1.10 ± 0.09 kg), 23 weeks old, were assigned to a 3 × 2 factorial design with 6 replicates pens containing 18 ducks and each duck was housed in one cage [18 ducks (cages)/ replicate]. Ducks were raised at 24°C and 74% RH during the experimental period. In addition, a 17-h photoperiod per d was adopted. The basal control diet met the requirements of the breed (Table 1). L-Threonine (L-Thr, 98.5%, ThreAMINO, Evonik Industries AG, Essen, Germany) was supplemented to the control diet at 0, 0.7, and 1.4 g /kg, corresponding 3.9, 4.6, and 5.3 g total Thr /kg with or without *B. subtilis* strain DSM 32315 0.0 and 0.5 g/kg, *B. subtilis* strain DSM32315 according to the manufacturer’s recommendation [min. 2 × 10^9^ CFU/g (Gut Care® PY1; Evonik Industries AG, Essen, Germany)]. The dietary amino acids were analyzed using HITACHIL-8900 (Hitachi, Ltd., Tokyo, Japan) (Table 2) (13). The Animal Care Committee of Guangdong Academy of Agriculture Sciences (Guangdong, PRC) approved the study.

**Table 1:** Composition and nutrient levels of the control basal diet.

Ingredients	Kg
Yellow Corn	325
Wheat	300
Peanut meal	130
Wheat bran	121
Limestone	84
Di-calcium phosphate	12
DL-Met	2.5
L-Lys	5
L-Trp	0.5
L-Ile	1.5
L-Val	1.5
L-Thr	0.0
Zeolite carrier	3.5
Salt	3.5
Premix^1^	10
Total	1,000
Nutrient levels, %	
ME (MJ/kg)	10.46
Crude protein	15.9
Calcium	3.5
Methionine	0.42
Lysine	0.89

^1^Provided per kilogram of diet: Vitamins: A, 12500 IU; D3, 4,125 IU; E, 15 IU; K, 2 mg; B1 1 mg; B2 8.5 mg; calcium pantothenate 50 mg; niacin 32.5 mg; pyridoxine 8 mg; biotin 2 mg; folic acid 5 mg; VB12 5 mg. Minerals: Zn 90 mg; I 0.5 mg; Fe 60 mg; Cu 8 mg; Se 0.2 mg; Co 0.26 mg; choline chloride 500 mg.

**Table 2:** Analyzed levels of amino acid in the experimental diets (3 × 2).^1^

Items, %	Treatments					
	*- Bacillus subtilis (0.0 g/kg)*			+ *Bacillus subtilis (0.5 g/kg)*		
	L-Thr supplementation (g/kg)			L-Thr supplementation (g/kg)		
L-Thr supplementation, g/kg	0.0	0.7	1.4	0.0	0.7	1.4
Essential amino acid						
Arginine	1.39	1.5	1.33	1.44	1.45	1.44
Histidine	0.35	0.33	0.33	0.34	0.35	0.35
Isoleucine	0.64	0.60	0.62	0.64	0.70	0.64
Leucine	1.06	1.02	1.01	1.06	1.08	1.10
Lysine	1.11	0.99	1.00	1.04	0.99	1.00
Methionine	0.32	0.30	0.34	0.35	0.34	0.43
Phenylalanine	0.72	0.70	0.70	0.71	0.73	0.74
Threonine	0.41	0.45	0.53	0.42	0.46	0.54
Valine	0.81	0.76	0.76	0.80	0.79	0.83
Tyrosine	0.52	0.50	0.50	0.55	0.55	0.56
Nonessential amino acid						
Alanine	0.69	0.62	0.64	0.66	0.67	0.69
Aspartic acid	1.43	1.35	1.30	1.39	1.37	1.39
Glutamic acid	3.32	3.30	3.31	3.344	3.53	3.55
Glycine	0.78	0.74	0.75	0.79	0.79	0.80
Proline	0.90	0.90	0.90	0.91	0.94	0.95
Serine	0.67	0.63	0.63	0.67	0.66	0.67

^1^Values are the results of a chemical analysis conducted in duplicate. Thr, Threonine.

### 2.2. Laying performance, productive performance, and egg quality

The number of eggs and feed intake for each replicate were recorded in order to calculate egg production and the feed conversion ratio (g feed intake/g egg produced). Each female breeder was inseminated (artificially) twice weekly with 100 L of pooled sperm at the age of 40 weeks. Fifty eggs from each replicate (*n* = 300 eggs per treatment) were incubated using an egg incubator under (Dezhou Jingxiang Technology Co, Dezhou, China) at 37.2°C to 38.0°C and 60 to 75% RH for 28 day. The fertility, hatchability, and ducklings’ weights were determined. Three eggs per replicate (*n* = 18 eggs per treatment) were collected at the end of the experiment to determine egg quality, as described by Ruan et al. (14). In addition, both albumen and yolk were extracted and kept at −20°C to determine amino acid content.

### 2.3. Amino acid analysis of egg albumen and yolk

The albumen and yolk samples were dried to constant weight and then defatted and hydrolyzed. An amino acid analyzer (HITACHIL-8900, Hitachi, Ltd., Tokyo, Japan) was used according to the AOAC method 994.12 (15). Briefly, a hundred milligrams of each sample were mixed with 800 μLof 0.1 MHCl and the oxygen was expelled bypassing nitrogen into the ampoule. Then, 800 μL of hexane was added and mixed by vertexing for1min, and centrifuged at 22,000 × g for 5 min, and the lower layer was collected. After acid hydrolysis with 6 NHCl at 110°C for 20 h, the amino acid contents were measured, except for cysteine and methionine. Methionine and cystine were measured by acid oxidation before hydrolysis with 6 NHCl (16). The absolute concentration was determined from the peaks of the sample and standard by comparing the peak area obtained for each amino acid with the peak area of the amino acid standards.

### 2.4. Early post-hatch performance of ducklings

At hatch, the total number of ducklings (*n* = 468) were assigned to the same dietary treatment groups (3 × 2 factorial design) for early post-hatch performance of ducklings. Each treatment group included six replicates of 13 ducklings. Ducklings were provided the same starter diets (Supplementary Table S1). Pens were supplied with water and feeders to provide *ad libitum* access to feed. On day 7 of age, body weight and feed consumption were determined, and mortality rates were recorded daily.

### 2.5. Statistical analysis

The data were analyzed with SPSS16.0 using a 3 × 2 factorial experimental design that included 2 factors: L-Thr (0, 0.7, and 1.4 g/kg) and *B. subtilis* strain DSM 32315 (0.0 and 0.5 g/kg) in order to determine the effects of dietary Thr and probiotic *B. subtilis* and their interaction on the reproductive performance of laying duck breeders, the amino acid composition of eggs, and early post-hatch duckling performance. A two-way ANOVA (SPSS 16; SPSS Inc., Chicago, IL) and Tukey’s multiple comparisons were performed to compare means. The a and b superscripts refer to a significant difference (*p* < 0.05), and * indicates a tendential difference between treatments at a specific time point (*p* = 0.051 ~ 0.06).

## 3. Results

### 3.1. Productive performance

The productive performance data are shown in Table 3. Increasing the L-Thr levels increased egg production rate compared with those of birds fed the control diet (66.22 ± 0.02 (0.7 g Thr /kg) and 66.14 ± 0.02% (1.4 g Thr /kg) *VS* 54.78 ± 0.02% (0.0 g Thr /kg); *p* < 0.05). The addition of *B. subtilis* did not affect the laying performance. Thr and probiotic (*B. subtilis*) co-supplementation had no effect on laying performance (*p* > 0.05).

**Table 3:** Effect of dietary L-Thr levels and *Bacillus subtilis* addition on laying performance of laying duck breeders from week 23 to 48 of age^1^.

Probiotic (*Bacillus subtilis*) g/kg	L- Thr (g/kg)	Egg production (%)	ADFI (g/d/bird)	Egg weight (g)	Egg mass (g/d)	FCR (g:g)
0.0	0.0	55.45	181	68.93	38.27	4.79
	0.7	65.83	177	73.17	48.65	3.84
	1.4	61.17	183	65.75	40.47	4.64
0.5	0.0	54.11	179	65.71	36.32	5.46
	0.7	66.61	184	73.21	49.67	4.04
	1.4	71.11	177	72.28	51.78	3.53
SEM		4.31	3.89	3.44	4.91	0.52
*p*-value						
Thr		0.02	0.97	0.23	0.06*	0.06*
*Bacillus subtilis*		0.38	0.86	0.69	0.39	0.85
*Bacillus subtilis* X Thr		0.39	0.26	0.36	0.38	0.22

^1^*n* = 6 replicates/treatment of 18 duck breeders per replicate. SEM, Standard error of mean. *indicates a tendential difference between treatments at a specific time point (*p* = 0.051 ~ 0.06). Thr, Threonine; ADFI, average daily feed intake; FCR, feed conversion ratio (feed intake/weight gain).

### 3.2. Egg quality

The results of egg quality are depicted in Table 4. Both Thr and *B. subtilis* demonstrated no significant effect on (*p* > 0.05) albumen height, Haugh unit, eggshell thickness, eggshell strength, or percentage of albumen, yolk, and eggshell.

**Table 4:** Effect of dietary L-Thr levels and *Bacillus subtilis* addition on egg quality at 48-week-old age.^1^

Probiotic (*Bacillus subtilis*) *g/kg*	L- Thr (g/kg)	Albumen Height (mm)	Haugh unit	Eggshell thickness (mm)	Eggshell strength (kgf)	Albumen (%)	Yolk (%)	Eggshell (%)
0.0	0.0	6.06	75.04	0.32	3.41	58.552	32.534	8.91
	0.7	5.84	75.51	0.332	3.76	59.482	31.2	9.31
	1.4	5.89	76.64	0.328	3.67	59.01	31.749	9.24
0.5	0.0	5.71	75.05	0.318	3.88	59.26	31.25	9.49
	0.7	5.40	71.28	0.321	3.72	58.428	32.556	9.01
	1.4	5.72	71.69	0.33	3.67	58.195	32.68	9.12
SEM		0.29	2.67	0.007	0.213	0.646	0.628	0.187
*p* value								
Thr		0.65	0.83	0.34	0.88	0.84	0.83	0.98
*Bacillus subtilis*		0.18	0.17	0.48	0.41	0.45	0.51	0.73
*Bacillus subtilis* X Thr		0.89	0.62	0.61	0.40	0.34	0.08	0.051*

^1^*n* = 3 eggs per replicate (*n* = 18 eggs per treatment). SEM, Standard error of mean. *indicates a tendential difference between treatments at a specific time point (*p* = 0.051 ~ 0.06).

### 3.3. Amino acid composition of egg

The amino acid composition results of egg albumen and yolk are displayed in Tables 5, 6. Except for proline, the addition of *B. subtilis* decreased (*p* < 0.05) the concentrations of amino acids in the egg white (albumen). *B. subtilis* supplementation decreased the concentrations of methionine, isoleucine, valine, and glycine while increasing the concentrations of cysteine, tyrosine, and serine (*p* < 0.05) in the egg yolk. In contrast, there was no significant influence (*p* > 0.05) of the dietary L-Thr on the amino acid concentrations in egg albumen and egg yolk.

**Table 5:** Effect of dietary L-Thr levels and *Bacillus subtilis* addition on amino acid composition (g/100 CP) of albumen in laying duck breeders^1^.

Amino acid	Treatments							SEM	*p*-Value		
	- *Bacillus subtilis* (0.0 g/kg)				+ *Bacillus subtilis* (0.5 g/kg)				Thr	*Bacillus*	*Bacillus subtilis* × Thr
	L-Thr supplementation (g/kg)				L-Thr supplementation (g/kg)						
	0	0.7	1.4		0	0.7	1.4				
Essential amino acid											
Arginine	3.62	3.59	3.57		3.35	3.33	3.33	0.043	0.70	<0.001	0.93
Isoleucine	2.52	2.43	2.40		2.29	2.27	2.29	0.041	0.30	<0.001	0.36
Leucine	5.85	5.83	5.78		5.43	5.38	5.43	0.068	0.84	<0.001	0.74
Lysine	5.31	5.31	5.30		4.99	4.94	4.97	0.062	0.94	<0.001	0.89
Methionine	4.09	4.11	4.29		3.92	3.87	3.72	0.096	0.99	<0.001	0.09
Phenylalanine	5.87	5.87	5.80		5.44	5.46	5.54	0.070	0.98	<0.001	0.38
Threonine	5.14	5.12	5.11		4.79	4.76	4.82	0.058	0.89	<0.001	0.80
Valine	4.27	4.13	4.12		3.91	3.88	3.93	0.058	0.31	<0.001	0.38
Histidine	1.59	1.58	1.57		1.44	1.43	1.45	0.018	0.91	<0.001	0.83
Semi-essential amino acid											
Cysteine	2.02	1.97	2.00		1.84	1.74	1.71	0.048	0.21	<0.001	0.581
Tyrosine	3.23	3.22	3.25		3.02	2.97	3.00	0.046	0.75	<0.001	0.803
Non- essential AAs											
Alanine	3.94	3.89	3.89		3.64	3.62	3.62	0.041	0.65	<0.001	0.91
Aspartic acid	7.84	7.88	7.87		7.40	7.34	7.40	0.091	0.95	<0.001	0.83
Glutamic acid	11.83	11.82	11.76		11.02	10.97	11.10	0.125	0.95	<0.001	0.71
Glycine	2.98	2.98	2.96		2.75	2.70	2.71	0.032	0.59	<0.001	0.69
Serine	7.04	7.03	7.01		6.58	6.58	6.62	0.077	0.99	<0.001	0.89
Proline	3.10	3.13	3.20		3.06	3.09	3.13	0.042	0.17	0.13	0.95

*n* = 18 egg albumen per replicate. SEM, Standard error of mean; Thr, Threonine.

**Table 6:** Effect of dietary L-Thr levels and *Bacillus subtilis* addition on amino acid composition (g/100 CP) of yolk in laying duck breeders^1^.

Amino acid	Treatments						SEM	*P*-Value		
	- *Bacillus subtilis* (0.0 g/kg)			+ *Bacillus subtilis* (0.5 g/kg)				Thr	*Bacillus*	*Bacillus subtilis* × Thr
	L-Thr supplementation (g/kg)			L-Thr supplementation (g/kg)						
	0.0	0.7	1.4	0.0	0.7	1.4				
Essential amino acid										
Arginine	1.898	1.881	1.828	1.820	1.817	1.829	0.086	0.93	0.51	0.88
Isoleucine	1.283	1.556	1.421	1.044	1.071	1.096	0.118	0.44	0.001	0.57
Leucine	2.274	2.540	2.417	2.279	2.279	2.293	0.150	0.67	0.30	0.67
Lysine	2.149	2.381	2.272	2.161	2.162	2.183	0.138	0.69	0.38	0.70
Methionine	0.580	0.548	0.521	0.485	0.314	0.328	0.057	0.12	0.001	0.46
Phenylalanine	1.248	1.374	1.322	1.244	1.214	1.232	0.072	0.79	0.16	0.56
Threonine	1.316	1.504	1.415	1.507	1.500	1.514	0.084	0.56	0.17	0.52
Valine	1.448	1.688	1.550	1.163	1.202	1.246	0.117	0.48	0.001	0.64
Histidine	0.790	0.835	0.809	0.749	0.746	0.759	0.045	0.89	0.11	0.85
Semi-essential amino acid										
Cysteine	0.256	0.233	0.213	0.366	0.402	0.455	0.029	0.71	<0.001	0.09
Tyrosine	0.882	0.862	0.861	1.136	1.148	1.139	0.064	0.99	<0.001	0.96
Non- essential AAs										
Alanine	1.520	1.450	1.372	1.408	1.428	1.445	0.060	0.65	0.68	0.31
Aspartic acid	2.493	2.640	2.525	2.560	2.545	2.549	0.128	0.85	0.98	0.80
Glutamic acid	3.652	3.623	3.436	3.436	3.417	3.450	0.157	0.80	0.29	0.71
Glycine	1.219	0.895	0.855	0.859	0.845	0.851	0.081	0.054*	0.045	0.07
Serine	1.695	2.001	1.892	2.372	2.366	2.372	0.123	0.47	<0.001	0.44
Proline	1.125	1.083	1.076	1.112	1.156	1.102	0.055	0.82	0.53	0.73

*n* = 18 egg yolk per replicate. SEM, Standard error of mean; Thr, Threonine. *indicates a tendential difference between treatments at a specific time point (*p* = 0.051 ~ 0.06).

### 3.4. Reproductive performance

The results of fertility and hatchability are presented in Table 7. Both Thr and *B. subtilis* demonstrated no significant effect on the reproductive performance (*p* > 0.05). The addition of *B. subtilis* without L-Thr decreased (*p* < 0.05) the percentage of egg hatchability compared with those of ducks fed diets of Thr at 0.7 and 1.4 g /kg along with dietary *B. subtilis* (*p* < 0.001).

**Table 7:** Effect of dietary L-Thr levels and *Bacillus subtilis* addition on fertility and hatchability rate in laying duck breeders.^1^

Probiotic (*Bacillus subtilis*) g/Kg	L- Thr (g/kg)	Fertility (%)	Hatchability of fertile eggs(%)	Mortality, %
0.0	0.0	80.97	67.60^ab^	0.048
	0.7	86.64	64.25^ab^	0.035
	1.4	84.61	69.72^ab^	0.037
0.5	0.0	83.99	54.16^b^	0.062
	0.7	83.31	71.76^a^	0.052
	1.4	86.02	64.62^ab^	0.042
SEM		3.74	3.72	
*P*-value				
Thr		0.71	0.13	0.61
*Bacillus subtilis*		0.90	0.23	0.39
*Bacillus subtilis* X Thr		0.68	0.02	0.93

*n* = 50 eggs per replicate. SEM, Standard error of mean; Thr, Threonine. Means sharing different letters (superscripts a, b) differ significantly.

### 3.5. Early post-hatch performance of ducklings

The findings of the early growth performance of ducklings are presented in Table 8. Increasing the L-Thr levels increased the hatching weight of ducklings compared with those of birds fed the control diet [33.98 ± 0.054 (0.7 g Thr /kg) and 34.17 ± 0.054 (1.4 g Thr /kg) *VS* 32.86 ± 0.054 g (0.0 g Thr /kg); *p* < 0.001]. In addition, the hatching weight of ducklings was higher in birds fed L-Thr supplementation at 1.4 g /kg compared to those of birds fed L-Thr supplementation at 0.7 g /kg (34.17 ± 0.054 vs. 33.98 ± 0.054 g, *p* < 0.001). In contrast, supplementation of *B. subtilis* decreased duckling hatching weight (*p* < 0 0.001). The addition of *B. subtilis* without L-Thr decreased hatching weight compared with those of ducks fed dietary Thr, along with dietary *B. subtilis* [31.95 ± 0.072 (0.0 g Thr /kg + 0.5 g *B. subtilis /kg*) vs. 34.08 ± 0.072 (0.7 g Thr /kg + 0.5 g *B. subtilis /kg*) and 33.98 ± 0.072 g (1.4 g Thr /kg + 0.5 g *B. subtilis /kg*); *p* < 0.001].

**Table 8:** Effect of dietary L-Thr levels and *Bacillus subtilis* addition on early post-hatch performance of ducklings.

Probiotic (*Bacillus subtilis*) g/Kg	L- Thr (g/kg)	Progeny early growth performance (ducklings)					
		Hatching weight, g (d 1)	BW, g (d7)	BWG, g	ADFI (g/d/bird)	FCR (g: g)	Mortality, %
0.0	0.0	33.78^b^	135	101	169	1.67	0
	0.7	33.89^b^	128	94.125	153	1.68	0
	1.4	34.36^a^	128	93.256	139	1.49	0
0.5	0.0	31.95^c^	127	94.733	125	1.31	0
	0.7	34.08^ab^	134	99.872	138	1.38	0
	1.4	33.98^b^	132	97.619	149	1.53	0
SEM		0.072	3.431	3.43	13.47	0.147	-
Thr		<0.001	0.90	0.76	0.96	0.97	-
*Bacillus subtilis*		<0.001	0.85	0.67	0.13	0.09	-
*Bacillus subtilis* X Thr		<0.001	0.09	0.16	0.15	0.35	-

*n* = 6 replicates/ treatment of 13 ducklings per replicate. SEM, Standard error of mean; Thr, Threonine; BW, body weight; BWG, body weight gain; ADFI, average daily feed intake; FCR, feed conversion ratio (feed intake/weight gain). Means sharing different letters (superscripts a-c) differ significantly.

## 4. Discussion

According to our knowledge, this is the first study to examine the simultaneous effects of Thr and *B. subtilis* supplementation on laying duck breeders. Following the restriction of antibiotics as growth promoters in many countries, the use of probiotics or synthetic amino acids in poultry diets has significantly increased (17–20). Previous studies found that a combination of amino acids and probiotics could synergistically or additively compensate to improve production performance, egg quality, and immunity (9). Therefore, we hypothesized that the combination of Thr and *B. subtilis* DSM32315 may improve the amino acid profile of eggs and the early post-hatch performance of ducklings fed antibiotic-free diets. In the present study, increasing dietary Thr intake improved egg production. It is well known that Thr is an indispensable amino acid because it cannot be synthesized in the body. In laying breeds, it has been reported that increasing dietary Thr level enhanced egg production, egg weight, egg mass, feed conversion ratio, albumen weight, and Haugh units (10, 21). Threonine has been shown to have a beneficial impact on nutrient absorption by augmenting the surface area of the villi (10). This may help to elucidate why the supplementation of Thr has been observed to enhance productive performance. Also, previous studies have reported an improvement in egg production in birds fed probiotics *B. subtilis* (5, 22, 23). However, in the present study, the addition of *B. subtilis* did not affect the laying performance. There is a scarcity of experiments on the effect of *B. subtilis* on laying ducks. Consistent with the findings of the present studies, the prior study revealed that probiotic supplementation (*Clostridium butyricum* and a combination of *Saccharomyces boulardii* and *Pediococcus acidilactici*) (24) or *B. subtilis* RX7 and B2A supplementation did not impact the laying rate under no stress conditions. Other studies have reported an improvement in egg production in birds fed probiotics *B. subtilis* (5, 22) under no stress conditions. These contradictory results may be attributed to differences in the addition level, diet composition, species and concentrations of *B. subtilis*, age of birds, different microorganisms used, and the experiential conditions (25). Since the study was conducted under normal conditions, *B. subtilis* DSM32315 has no effect on laying performance. Further studies are needed to evaluate the effects of *B. subtilis* DSM32315 on breeder-laying ducks under different stress conditions.

The current study showed that Thr supplementation had no effects on the egg quality parameters. However, eggshell percentage increased after supplementing a combination of *B. subtilis* DSM32315 and Thr. Studies have shown that supplementing laying hens with probiotics improves eggshell quality and decreases the number of damaged eggs (26, 27). Inclusion of *B. subtilis* in hen laying hen diets improved eggshell quality as a result of enhancing calcium absorption (28). Ovalbumin is the predominant protein in albumen; it provides essential amino acids for chicken embryo development and is a valuable source of amino acids required for embryonic development (29). Herein, the addition of *B. subtilis* decreased methionine, isoleucine, and valine, in the egg albumen and yolk. This finding indicates that *B. subtilis* may affect essential amino acids and limit their retention and decreasing embryonic growth rates.

In breeding programs and production, the hatchability rate is an essential factor. Intriguingly, the percentage of ducklings that successfully hatched and their hatching weights increased when birds were fed *B. subtilis* with 0.7 g/kg Thr. Daris et al. (26) suggested that dietary *B. subtilis* improve eggshell quality, which led to enhance hatchability in broiler breeder hens.

## 5. Conclusion

The current study revealed that the combination supplemented of L-Thr and *B. subtilis* DSM 32315 is recommended due to its positive effects on the eggshell percentage, hatchability and the body weights of newly hatched ducklings when dietary Thr was added at a rate of 0.7 g/kg and *B. subtilis* DSM 32315 at 0.5 g/kg. In addition, adding L-Thr separately at 0.7 g/kg could improve the egg production of duck breeders. Further studies are required to find the proper dosages of *B. subtilis* DSM 32315 with co-dietary inclusion of limiting amino acids in the diets of duck breeders. The findings of these trials will support feed additive interventions to transition into antibiotic-free diets.

## Data availability statement

The raw data supporting the conclusions of this article will be made available by the authors, without undue reservation.

## Ethics statement

The Animal Care and Use Committee of Guangdong Academy of Agriculture Sciences approved all experimental procedures (GAASIAS-2019-020).

## Author contributions

MA and CZ: conceptualization. MA, HE-S and CZ: methodology. MA: investigation and writing–original draft preparation. WX and SW: data curation. YZ, WC, and WX: formal analysis. MA and HE-S: writing–review and editing. CZ: supervision. CZ and MA: funding acquisition. All of the authors contributed to the article and approved the final version.

## Funding

This study was supported by the China Agriculture Research System of MOF and MARA (Grant No. CARS-42-13), the Modern Agricultural Industry Technology System Innovation Team of Guangdong Province(Grant No. 2019KJ137), the Special Fund for Scientific Innovation Strategy-Construction of High Level Academy of Agricultural Sciences (R2020PY-JX008, 202106TD), and the Science and Technology Program of Guangdong Province (Grant No. 2019A050505007, 2021A0505050003), Foreign Expert Project (QNL20200130001) and Researchers Supporting Project Number (RSPD2023R731), King Saud University (Riyadh, Saudi Arabia).

## Conflict of interest

The authors declare that the research was conducted in the absence of any commercial or financial relationships that could be construed as a potential conflict of interest.

## Publisher’s note

All claims expressed in this article are solely those of the authors and do not necessarily represent those of their affiliated organizations, or those of the publisher, the editors and the reviewers. Any product that may be evaluated in this article, or claim that may be made by its manufacturer, is not guaranteed or endorsed by the publisher.
