# Supplementary material for: The impact of Bacillus subtilis DSM32315 and L-Threonine supplementation on the amino acid composition of eggs and early post-hatch performance of ducklings
Source: Front Vet Sci. 2023 Aug 23;10:1238070. doi: 10.3389/fvets.2023.1238070 (PMC10481339; doi:10.3389/fvets.2023.1238070)
Supplement: Supplementary file 1 [file Table_1.doc]

***Supplementary Material***

**The impact of *Bacillus Subtilis* DSM32315 and threonine supplementation on the amino acid composition of eggs and early post-hatch performance of ducklings**

**Mahmoud Mostafa Azzam^1^, Wei Chen^2^, Weiguang Xia^2^, Shuang Wang^2^, Yanan Zhang^2^, HebatAllah Kasem El‐Senousey^2,3^, and** **Chuntian Zheng^2*^**

***Corresponding Author:** [zhengchuntian@gdaas.cn](mailto:zhengchuntian@gdaas.cn)

**Composition and nutrient levels in the experimental diets of ducklings age from hatchling to 7 d of age.**

| Ingredient | % |
| --- | --- |
| Corn, % | 61.90 |
| Soybean meal, % | 27.8 |
| Wheat bran, % | 6.40 |
| Limestone, % | 0.77 |
| Di-calcium phosphate, % | 1.60 |
| Salt, % | 0.30 |
| Lysine, % | 0.05 |
| DL- Methionine, % | 0.18 |
| Premix^1^, % | 1.00 |
| Total, % | 100.0 |
| Calculated chemical composition (as fed basis) | |
| Metabolizable energy, kcal/kg | 2800 |
| Crude protein, % | 19 |
| Calcium, % | 0.85 |
| Total phosphorus, % | 0.66 |
| Non-phytate phosphorus, % | 0.4 |
| Lysine, % | 1.05 |
| Methionine, % | 0.45 |
| Methionine + Cystine, % | 0.76 |
| Threonine, % | 0.7 |
| Tryptophan, % | 0.22 |
| Arginine, % | 1.25 |

^1^The premix provided the following per kilogram of diet: vitamin A 5,500 IU, vitamin D3 400 IU, vitamin E 10 IU, vitamin K 2.0 mg, vitamin B1 3.0 mg, vitamin B2 4.6 mg, vitamin B6 2.2 mg, vitamin B12 0.02 mg, choline 500 mg, D-calcium pantothenate 7.4 mg, folic acid 1.0 mg, biotin 0.08 mg, Fe 80 mg, Cu 10 mg, Mn 39 mg, Zn 52 mg, I 0.26 mg, and Se 0.15 mg.
